# Supplementary material for: Cancer detection for small-size and ambiguous tumors based on semantic FPN and transformer
Source: PLoS One. 2023 Feb 16;18(2):e0275194. doi: 10.1371/journal.pone.0275194 (PMC9934456; doi:10.1371/journal.pone.0275194)
Supplement: Attachment — Submitted filename: Review_PLOS4.docx [file pone.0275194.s001.docx]

1. Kindly write the abstract is a concise and succinct manner
2. In abstract SPN-TS, CBIS-DDSM full form missed
3. In Table 1 citation missed
4. *et al.* should be in italics
5. Please avoid the word “we” throughout the script
6. MCC full form in the abstract?
7. Exact motivation of the work in the introduction missed
8. Learning rate of the network?
9. Tuning of the network was missed
10. Please remove unnecessary capitalizations in the manuscript
11. Please mention important major contributions only
12. Quality of the images are very poor, and not visible also
13. Needs to include ROC curves, and precision-recall curves
14. Grammatically needs to recheck again
